# Mobilizing for a war on the home front against sugar-related morbidity and mortality

**DOI:** 10.1186/s13584-017-0155-2

**Published:** 2017-05-31

**Authors:** Dean Schillinger, James G. Kahn

**Affiliations:** 1 Division of General Internal Medicine and Center for Vulnerable Populations, University of California San Francisco and Zuckerberg San Francisco General Hospital, San Francisco, CA USA; 2 Philip R. Lee Institute for Health Policy Studies, University of California San Francisco, San Francisco, CA USA

## Abstract

In Israel today, there are 420,200 Israelis diagnosed with diabetes, and every year, Israelis sustain thousands of diabetes-related deaths and tens of thousands of diabetes-related amputations. As such, in Israel, as in much of the world, there is a silent and deadly public health war against obesity and diabetes taking place on the home front -- one in which clinicians, patients, and families fight thousands of life- and limb-threatening battles daily, involving preventable heart disease, diabetes, strokes and amputations. Yet the global clinical and scientific communities, indeed society at large, have barely begun to mobilize. Fighting this war requires confronting and altering “obesogenic” and “diabetogenic” economic and social factors, including food and beverage marketing and pricing that push diets engorged with processed sugars. Ginsberg, in a study recently published in IJHPR, contributes to our understanding of the combined sugar-related health burdens in Israel, producing an epidemiology and health economics study that estimates the health burdens of obesity, overweight, and dental caries in Israel today. He projects the reductions resulting from that portion of disease burden and associated costs if sugar consumption declined to 10 or 5% of daily caloric consumption as a result of multifaceted public health interventions. Projected over 70 years, these reductions in sugar consumption would prevent 16,590 and 34,580 deaths, respectively. These numbers of Israeli deaths *averted* are similar to, or exceed, the total resulting from armed conflict or terrorism over the past 70 years. While overconsumption of sugar is only one of many factors that drive cardio-metabolic disease, the study by Ginsberg suggests a path through which we can overcome the numerous internal and external obstacles that societies face in making a public policy commitment to fight the warm on the home front: promoting health by reducing added sugar exposure.

## Background

During the Arab-Israel conflict, from the founding of the state of Israel in 1948 to the present day, 14,408 Israeli Defense Force soldiers have died in combat [1] and 3058 Israeli civilians have died from terrorist acts [2]. Together, these 17,466 deaths over nearly 70 years yield an average of 253 deaths per year. Understandably, these 253 deaths a year hold a prominent place in the hearts and minds of all Israelis. Yet, in Israel today, there are 420,200 Israelis diagnosed with diabetes [3], comprising over 7% of its population, and every year, Israelis sustain thousands of diabetes-related deaths and tens of thousands of diabetes-related amputations.

As such, in Israel, as in much of the world, there is a more silent and deadly public health war on the home front -- one in which clinicians, patients, and families fight thousands of life- and limb-threatening battles daily, involving preventable heart disease, diabetes, strokes and amputations. This is the public health war against obesity and diabetes. Yet the global clinical and scientific communities, indeed society at large, have barely begun to mobilize. Fighting this war requires confronting and altering “obesogenic” and “diabetogenic” economic and social factors – characterized by sedentary work and lifestyles, food and beverage marketing and pricing that push diets engorged with processed sugars, and neighborhoods in which it is far easier and safer to drive than to walk or bike.

Ginsberg [4] contributes importantly to our understanding of the combined sugar-related health burdens in Israel, producing an epidemiology and health economics study that estimates the health burdens of obesity, overweight, and dental caries in Israel today. He projects the reductions resulting from that portion of disease burden and associated costs if sugar consumption declined as a result of multifaceted public health interventions.

Ginsberg first calculates that in Israel there are 6402 deaths annually from overweight and obesity (15% of all deaths). These far exceed annual deaths from armed conflict and terrorism. He then projects that, were sugar consumption in Israel to drop from its current 12.45 to 10% of caloric intake (the upper threshold of WHO recommendation on added sugars), 237 deaths would be prevented annually. Were sugar consumption in Israel to drop to 5% (the more stringent WHO recommendation), 494 deaths would be prevented annually. Projected over 70 years, these reductions in sugar consumption would prevent 16,590 and 34,580 deaths, respectively. These numbers of Israeli deaths *averted* are similar to, or exceed, the total resulting from armed conflict or terrorism over the past 70 years. While some may argue that the comparison to younger Israelis in the IDF who perished is disingenuous, in that the sugar-related deaths disproportionately occur in older adults, two points must be made. First, Ginsberg finds that each preventable sugar-associated death reduces life expectancy by a striking 12.4 years. Second, a significant proportion of deaths in young and middle-aged adults are attributable to the consumption of sugar-sweetened beverages (SSBs) alone. For comparison, in the US, which has similar rates of SSB consumption to Israel, among adults less than 45 years old, 9.6% of women’s deaths and 12.5% of men’s deaths are attributable to SSBs [5].

Finally, from an economic standpoint, Ginsberg determines that a multifaceted intervention with a target sugar consumption of 10% of daily calories would yield significant costs savings, estimated at 301 million NIS per year, or 0.02% of GDP. A target consumption of 5% would yield even greater costs savings, estimated at 610 million NIS per year, or 0.03% of GDP.

Why are these findings important? We have previously written about the worrisome level of complacency among scientists, clinicians and the general public with respect to sugar-related diseases [6]. The underlying causes for this complacency are varied: misguided beliefs in the *individual* behavioral or genetic paradigms of disease causation, rather than multi-level socio-ecological forces; inappropriate cynicism and antipathy regarding the efficacy of public policy solutions; mistaken beliefs that sugar-related non-communicable diseases reflect the price of economic well-being; lack of a powerful and politically active advocacy community; absence of a “second-hand smoking gun” equivalent (the fact that passive smoking leads to morbidity and mortality); and aggressive interference, misinformation and counter-campaigns from the sugar and beverage industries [7]. However, recent political and social action in Mexico [8], the US, France and other countries and regions related to taxation on SSBs and warning labels [9] suggest that society is turning a corner, moving from one of complacency to early activation. That the WHO endorsed such taxes marks an important watershed in the fight against sugar-related diseases. The study by Ginsberg lends additional support for the imperative to mobilize in the war against sugar-related diseases, not just in Israel, but in all middle and high income countries.

Readers should keep in mind that in many respects, Ginsberg’s calculations regarding the burden of sugar-related diseases represent an underestimation. First, the only measures of the costs of disability were derived from medical care data. The direct costs of disability (e.g. related to ongoing disability after surviving an amputation or a stroke) can far exceed those reflected by direct medical costs. Second, the estimates on morbidity related to dental caries were derived only from children and youth; there are significant oral health consequences of sugar consumption in adults as well. Third, his estimates of the sugar-associated burden of cardiovascular and metabolic diseases were based on a conservative and faulty assumption that the relationships between sugar consumption and cardio-metabolic diseases are mediated solely by overweight and obesity. There is accumulating evidence [10] that added sugars, in particular excess fructose (which is half of the sucrose molecule, e.g. table sugar), leads directly to metabolic disturbances, such as insulin resistance and lipid abnormalities, that cause cardio-metabolic disease independent of body weight and often in the absence of obesity.

The “elephant in the room” is specifying just how to structure a multifaceted intervention that can reduce sugar consumption to 10 or 5% of daily caloric intake.. It *potentially* includes some or all of the following [4]: a gradual reduction of sugar content in everyday food and drink products, combined with reductions in portion size; price increases of at least 10–20% on high sugar products through the use of a tax such as on full sugar soft drinks; reduction in price promotions in all retail outlets including supermarkets, stores, restaurants and takeaways; reduction in advertisements for high sugar food and drink products to children and adults; implementation of public sector catering standards to ensure provision and sale of healthier food and drinks in hospitals, leisure centers; and providing practical steps to help individuals lower their own and their families’ sugar intake. With the exception of price increases, which appear to demonstrate fairly robust price elasticity, the other facets of the intervention have been inadequately studied, either alone or in combination.

Apparently, the Israeli Health Minister is reluctant to impose a sugar tax, on the grounds that such a tax will raise consumer prices in a “regressive fashion” [11]. The argument that sugar taxes are socially and economically regressive is one that is widely touted by the sugar and beverage industries in their anti-tax campaigns. However, this argument flies in the face of recent data suggesting that the economic and health *gains* of reducing sugar consumption are disproportionately awarded to those disproportionately exposed: children and socioeconomically deprived adults [9].

While overconsumption of sugar is only one of many factors that drive cardio-metabolic disease, the study by Ginsberg suggests a path through which we can overcome the numerous internal and external obstacles that societies face in making a public policy commitment to promote health by reducing added sugar exposure. The anti-tobacco movement evolved over 40 years, and involved several generations of clinician- and scientist-advocates. Reframing initiatives to limit sugar consumption as a war on the home front, one that requires step-wise efforts and long-term commitment, will be critical for engagement. We can and should develop new alliances across scientific, clinical, public health, business and lay communities, and create effective strategies that squarely confront the unhealthy social, economic, and environmental conditions that are the primary drivers of these cardio-metabolic epidemics. The contribution by Ginsberg provides a clear rationale for concerned scientists, public health practitioners and clinicians to become more active in affecting policy in their communities, their municipalities, and their nations. It is time for those of us fighting these epidemics through one small clinical battle at a time to enlist *en masse* in the larger and longer national and global policy war against sugar-associated diseases*.*

## Conclusions

While overconsumption of sugar is only one of many factors that drive cardio-metabolic disease, the study by Ginsberg suggests a path through which we can overcome the numerous internal and external obstacles that societies face in making a public policy commitment to promote health by reducing added sugar exposure. The anti-tobacco movement evolved over 40 years, and involved several generations of clinician- and scientist-advocates. Reframing initiatives to limit sugar consumption as a war on the home front, one that requires step-wise efforts and long-term commitment, will be critical for engagement. We can and should develop new alliances across scientific, clinical, public health, business and lay communities, and create effective strategies that squarely confront the unhealthy social, economic, and environmental conditions that are the primary drivers of these cardio-metabolic epidemics. The contribution by Ginsberg provides a clear rationale for concerned scientists, public health practitioners and clinicians to become more active in affecting policy in their communities, their municipalities, and their nations. It is time for those of us fighting these epidemics through one small clinical battle at a time to enlist *en masse* in the larger and longer national and global policy war against sugar-associated diseases.

## References

[1] http://www.jewishvirtuallibrary.org/total-casualties-arab-israeli-conflict. Accessed 16 Apr 2017.

[2] http://www.jewishvirtuallibrary.org/number-of-terrorism-fatalities-in-israel. Accessed 16 Apr 2017.

[3] http://www.idf.org/membership/eur/israel. Accessed 16 Apr 2017.

[4] Ginsberg GM (2017). Mortality, hospital days and treatment costs of current and reduced sugar consumption in Israel. Isr J Health Policy res.

[5] Singh et al. Global SSB-related morbidity and mortality. 2015 doi: 10.1161/CIRCULATIONAHA.114.010636.

[6] Schillinger D (2016). Confronting complacency in the face of calamity: mobilizing for the home front war against diabetes. J Endocrinol Diab.

[7] Schillinger D, Tran J, Mangurian C, Kearns C (2016). Do sugar-sweetened beverages cause obesity and diabetes? Industry and the manufacture of scientific controversy. Ann Intern Med.

[8] Arantxa Colchero M, Rivera-Donmarco J, Popkin B, Wen NS (2017). In Mexico, evidence of sustained consumer response two years after implementing a sugar-sweetened beverage tax. Health Aff March.

[9] Schillinger D, Jacobson MF (2016). Science and public health on trial: warning notices on advertisements for sugary drinks. JAMA.

[10] Lustig R. Sickenly sweet: does sugar cause Type 2 diabetes? Yes. Can J Diabetes. 2016;40(4):282–6. 10.1016/j.jcjd.2016.01.004 27216628

[11] Dovrat-Meseritz A. New Israeli health-labeling rules less strict than food makers feared. Haaretz (Newspaper). 2016. http://www.haaretz.com/israel-news/business/1.754454. Accessed 29 Dec 2016.

